# Mild cognitive impairment in presymptomatic amyotrophic lateral sclerosis

**DOI:** 10.1007/s00415-026-14206-0

**Published:** 2026-10-25

**Authors:** Caroline McHutchison, Joanne Wuu, Stephanie Cosentino, Regla Saldana, Enjolie Vadella, Ana Benacerraf, Katja McBane, Alexa S. Gonzalez, Jessica H. Stark, Yindi Li, Sharon Abrahams, Michael Benatar

**Affiliations:** 1 School of Psychology, Faculty of Natural Sciences, University of Stirling, 3B101 Cottrell Building, Stirling, FK9 4LQ UK; 2 Euan MacDonald Centre for MND Research, University of Edinburgh, Edinburgh, UK; 3 Department of Neurology and the ALS Center, University of Miami Miller School of Medicine, 1120 NW 14 Street, CRB1318, Miami, FL 33136 USA; 4 The Taub Institute for Research on Alzheimer’s Disease and the Aging Brain, Columbia University, New York, NY USA; 5 Department of Psychology, School of Philosophy, Psychology, and Language Sciences, University of Edinburgh, Edinburgh, UK

**Keywords:** Presymptomatic disease, Motor neuron disease, Frontotemporal dementia, Prodromal symptoms

## Abstract

**Background:**

Amyotrophic lateral sclerosis (ALS) and frontotemporal dementia (FTD) are clinically and biologically related, and each may be preceded by a prodromal stage of disease. Mild cognitive and behavioural impairment (MCI/MBI) have also been observed in those with clinically manifest ALS. The primary aim of this study was to characterise MCI in presymptomatic ALS.

**Methods:**

Criteria for frontotemporal-related MCI (MCI-ft), which require evidence of subthreshold cognitive performance or decline, were adapted from existing frameworks for MCI in prodromal FTD, presymptomatic ALS and clinically manifest ALS. MCI-ft was characterised in 216 *Pre-fALS* participants (74 *C9ORF72*, 94 *SOD1*, 20 other pathogenic variant carriers and 28 non-carriers) with neuropsychological data at 807 study visits. All designations were reviewed by a research neuropsychologist, with consensus discussions as needed.

**Results:**

MCI-ft was observed in 15 (20%) *C9ORF72*, 12 (13%) *SOD1* and 3 (%) of other carriers at one or more study visits, compared to 1 (4%) non-carrier. Most visits with MCI-ft showed impairment affecting both language and executive functioning. Fluctuations in MCI-ft status between assessments were common.

**Conclusion:**

The observed frequency and temporal course of MCI-ft in presymptomatic ALS, using criteria based on subthreshold cognitive performance and decline, expands understanding of this phase of disease. These findings help to inform future research examining the risk of phenoconversion to clinically manifest disease and aid the design of disease prevention trials.

**Supplementary Information:**

The online version contains supplementary material available at https://doi.org/10.1007/s00415-026-14206-0.

## Introduction

Amyotrophic lateral sclerosis (ALS) is characterised primarily by degeneration of upper and lower motor neurons. Cognitive and behavioural features, however, are common [1], with up to 10–15% of ALS patients also meeting criteria for frontotemporal dementia (ALS-FTD), most often the behavioural variant subtype (bvFTD). Cognitive domains typically affected include language, letter fluency, and executive functioning [1, 2]. Whilst apathy is the most common behavioural manifestation in ALS [1, 2], others from the bvFTD revised diagnostic criteria [3] as well as gregariousness, joviality, and loss of insight, have been reported in ALS-FTD [4], FTD [3, 5] and those at elevated genetic risk for FTD [5]. These non-motor symptoms are encompassed by the term “ALS frontotemporal spectrum disorder” (ALS-FTSD) in the context of clinically manifest ALS [2]. Their presence is associated with a reduced quality of life, increased caregiver burden [6], and compromised decision-making capacity regarding medical care and treatment [7].

The discovery of genetic causes of ALS and FTD has enabled the study of neuropsychological deficits during the presymptomatic stage of disease—where mild, clinically detectable signs may be present, but are insufficient to declare phenoconversion and diagnosis [8, 9]. For cognition, lower average letter fluency and task initiation scores have been reported in unaffected *C9ORF72* carriers compared to non-carrier controls [10–12]. In presymptomatic FTD, unaffected progranulin (*GRN*) and microtubule-associated protein tau (*MAPT*) carriers have also shown poorer performance on tests of language, executive functioning and verbal fluency [13]. On average, these carriers have shown declines in performance on tests of language, attention, executive functioning, social cognition and memory over a 4-year period [14]. However, the frequency of clinically relevant cognitive impairment in presymptomatic ALS is yet to be described. Some studies of people with ALS have identified cognitive deficits at first assessment [15, 16], suggesting that these symptoms arose either during the early stages of clinically manifest ALS or as part of a presymptomatic stage of ALS.

Several approaches have been proposed for defining mild cognitive or behavioural impairment (MCI/MBI) prior to the emergence of clinically manifest ALS or FTD (described in Supplementary Table 1). In ALS-FTSD, criteria have been proposed and revised [2] (henceforth referred to as “the Revised Strong Criteria”) but were not developed to delineate cognitive or behavioural impairment in the absence of ALS. Therefore, a separate conceptual framework for MCI/MBI in presymptomatic ALS was proposed following the 1st International Pre-symptomatic ALS Workshop in 2020 [9] which, like the Revised Strong Criteria, proposed separate delineation of MCI and MBI given that cognitive and behavioural impairment may occur independently [2].

The Miami Framework built upon these ideas, proposing separate axes to capture the motor neuron and frontotemporal phenotypic continua that range from clinically silent, to prodromal, to clinically manifest disease [17]. Progression along each axis is independent. MCI is envisioned as a prodromal stage along the frontotemporal axis that may or may not progress to clinically manifest FTD and might arise during the presymptomatic or clinically manifest stages of ALS. The primary goal of the current study was to characterise the prevalence and temporal pattern of MCI in presymptomatic ALS using data from the *Pre-symptomatic Familial ALS* (*Pre-fALS*) study.

## Methods

### Definitions of cognitive functioning

Common terms used to describe cognitive functioning have previously been defined differently but used interchangeably. Here we use “subthreshold cognitive performance (subTCP)” to describe scores on an objective test of cognition that fall below a specified cut-off compared to appropriate normative data; and “cognitive *decline*” to describe meaningful change in cognitive test scores compared to prior performance, also based on a specified cut-off. We use MCI-ft to denote the syndrome of mild cognitive impairment as a prodromal stage of the frontotemporal axis, as described by the Miami Framework [17], for those with presymptomatic and clinically manifest ALS and to refer to the criteria presented here, with MCI used to capture the general concept of mild cognitive impairment. See Box 1 for operational definitions.

**Box 1:** Definitions of terms related to cognitive functioning

Term	Definition
Subthreshold cognitive performance (SubTCP)	Scores on an objective test of cognitive functioning that fall below a specified cut-off, where the cut-off may be defined based on: A pre-specified unit(s) of standard deviation (e.g. 1.5 SD) below the mean of the appropriate normative data, OR A published raw score cut-off based on appropriate normative data
Cognitive decline	Worsening of cognitive functioning based on: Changes between prior and current scores on objective cognitive tests that exceed a specified cut-off, OR Self- or study-partner report
Mild cognitive impairment (MCI)	A clinical syndrome of cognitive impairment of any variety (frontotemporal, amnestic, global, etc.) that is insufficient to meet the criteria for a diagnosis of dementia
Frontotemporal-related mild cognitive impairment (MCI-ft)	A more specific term that refers to MCI of the frontotemporal variety and meeting the criteria described in this paper

### Study population

The ongoing *Pre-fALS* study has previously been described [8, 9]. Briefly, *Pre-fALS*, initiated in 2007, is a natural history and biomarker study that recruits individuals at elevated genetic risk for ALS or FTD (namely, carriers of pathogenic variants in any disease-associated gene, including *SOD1*, *C9ORF72, FUS, TARDBP, VCP, *etc*.*) from across North America. Participants undergo genetic counselling and testing prior to enrolment, with the opportunity to learn their genetic status (disclosure group) or not (non-disclosure group), if carrier status is not already known (known status group). The subset of participants in the non-disclosure group who are not carriers of the pathogenic variant(s) serve as controls and remain blinded to their genetic status. All participants are asymptomatic (i.e. do not have clinically manifest disease) at the time of enrolment.

As of data cut-off in May 2024, *Pre-fALS* had screened ~ 470 participants, with ~ 235 enrolled in the longitudinal *Pre-fALS* Cohort. All participants provided written informed consent.

### Study procedures

*Pre-fALS* participants are evaluated every ~ 12–36 months with multi-modal assessments including neuromuscular examination, electromyography, and other biomarker procedures. Cognition is assessed using both a comprehensive neuropsychological battery (since 2011) and the Edinburgh Cognitive and Behavioural ALS Screen (ECAS) [18, 19] (since 2015). The ECAS produces ALS-specific (executive functioning, language, and letter fluency), ALS non-specific (memory and visuospatial skills) and total scores. The North American English ECAS alternate versions and age- and education-adjusted norms [19] were used*.* The comprehensive neuropsychological battery has been adapted over the years, with the current battery (described elsewhere [9] and in Supplementary Table 2) evaluating a range of cognitive domains (executive functioning, social cognition, language, memory and visuospatial skills) and a measure of premorbid IQ. Tests were selected based on their sensitivity to subTCP in ALS and FTD, the availability of appropriate published normative data, minimal overlap with the cognitive functions assessed by the ECAS, and suitability for those with physical disability to facilitate continued assessment amongst those who phenoconvert to clinically manifest ALS. All neuropsychological assessments are conducted by trained research staff with ECAS certification.

### Characterising MCI-ft in Pre-fALS

Prior to characterising MCI in *Pre-fALS*, we: (1) compared the different published MCI/MBI criteria from the ALS and FTD communities (Supplementary Table 1); and (2) leveraged ideas and concepts from these published criteria to refine research criteria for MCI as a potentially prodromal stage of FTD (MCI-ft; see Supplementary Methods for full details) with MBI-ft to be addressed separately in a future publication. Considering MCI-ft and MBI-ft separately will allow better characterisation of neuropsychological profiles in presymptomatic ALS and enable future studies to determine whether MCI-ft and MBI-ft are differentially associated with phenoconversion to ALS versus FTD. These criteria may be combined at a later stage if supported by the data.

The MCI-ft Criteria were then used to identify MCI in all *Pre-fALS* participants with neuropsychological evaluation as of May 2024. Cognitive function was classified as FTD, MCI-ft, MCI-ft uncertain, unimpaired, or indeterminate (where cognitive data were incomplete). All MCI-ft classifications were reviewed by a research neuropsychologist (CM). Participants were selected for discussion at a consensus meeting involving a neurologist (MB) and additional neuropsychologist (SC) if, at a study visit, neuropsychological performance was poor, confounding factors were present, or the evaluator raised concerns about the participant’s performance. For all classifications, information available at the current assessment was considered, with relevant information from prior assessments also included where appropriate.

All analyses were conducted using R [20] version 4.5.0 (2025-04-11). Data are available from the corresponding author (MB) upon reasonable request.

## Results

Demographic details at initial neuropsychological assessment (henceforth referred to as baseline) are summarised in Table 1.

**Table 1:** Baseline characteristics

	*Pathogenic variant carriers*				Non-carriers^a^ (*N* = 28)	All participants (*N* = 216)
	*C9ORF72* (*N* = 74)	*SOD1* (*N* = 94)	Other genes (*N* = 20)	All carriers (*N* = 188)		
Age	45.4 ± 12.1	43.4 ± 12.3	44.8 ± 13.8	44.3 ± 12.4	45.4 ± 12.1	44.5 ± 12.3
Sex (male)	32 (43%)	33 (35%)	7 (35%)	72 (38%)	10 (36%)	82 (38%)
Education						
< College	23 (31%)	43 (46%)	9 (45%)	75 (40%)	10 (36%)	85 (39%)
≥ College	51 (69%)	51 (54%)	11 (55%)	113 (60%)	18 (64%)	131 (61%)
Number of visits with neuropsychological assessment^b^	4 [3, 5]	5 [2, 7]	2 [1, 2.5]	3 [2, 5]	3 [2, 4]	4 [2, 5]

Values represent mean ± SD or *n*(%) unless otherwise specified

Includes all *Pre-fALS* participants with neuropsychological data as of May 2024

^a^Gene negative, i.e. unaffected individuals without a pathogenic variant associated with ALS or FTD

^b^Median [*Q*1, *Q*3]

### MCI-ft Criteria

Divergent rates of cognitive impairment based on three of the published frameworks [2, 5, 9] were identified. The evaluation of key differences between published prodromal criteria in ALS-FTSD and FTD focussed on the cognitive domains to be included, the definition of decline from baseline and the role or premorbid IQ, subTCP in the absence of decline, and the role of letter fluency (described in full in the Supplementary Results and Supplementary Table 3). Findings informed the final MCI-ft Criteria (Table 2) where the presence of MCI-ft is designated based on:

- SubTCP (≥ 2 SD below the norm) at a single evaluation, or
- Decline (≥ 1.5 SD) from baseline assessment

**Table 2:** MCI-ft research criteria

*Guidance note:*
* Criteria are applied hierarchically, from top to bottom. Once the criteria for a given classification is met, stop*
* Decline requires data from at least 1 prior timepoint, whereas subthreshold cognitive performance (subTCP) does not*
* The number of test(s) stated in a criterion below indicates the minimal number of tests required*
*MCI-ft*^a^
At least 1 of the following based on objective cognitive assessment:
(A) Decline from baseline (≥ 1.5 SD reduction on adjusted *z* score) on 2 tests of executive functioning, social cognition, or language^b,c^
(B) SubTCP (adjusted *z* score ≥ 2^d^ below the norm) on 2 tests of executive functioning, social cognition, or language^b,e^
(C) Decline from baseline (≥ 1.5 SD reduction on adjusted *z* score) on 1 test of executive functioning, social cognition, or language AND subTCP (adjusted *z*-score ≥ 2^d^ below the norm) on 1 other test of executive functioning, social cognition, or language^b,c,e,f^
*MCI-ft uncertain*
At least 1 of the following based on objective cognitive assessment:
(A) Decline from baseline (≥ 1.5 SD reduction on adjusted *z* score) on 1 test of executive functioning, social cognition, or language^c^
(B) SubTCP (adjusted *z* score ≥ 2^d^ below the norm) on 1 test of executive functioning, social cognition, or language^e^
(C) SubTCP (adjusted *z* score ≥ 1.5^g^ below the norm) without evidence of decline from baseline (≥ 1.5 SD reduction on adjusted *z* score) on 2 tests of executive functioning, social cognition, or language^b,e^
(D) Decline from estimated premorbid IQ (≥ 1.5 SD reduction on adjusted *z* score relative to the adjusted *z* score of estimated premorbid IQ^h^) on 2 tests of executive functioning, social cognition, or language^b^
OR
(A) Self- or study partner-reported decline in executive functioning, social cognition, or language
*MCI-non-ft*
(A) Meets published criteria for mild cognitive impairment related to a neurodegenerative disease other than FTD (e.g. Alzheimer’s-related MCI)
*Indeterminate*
(A) Incomplete assessment^i^ or no cognitive data available
*Unimpaired*
Both of the following based on objective cognitive assessment:
(A) SubTCP (adjusted *z* score ≥ 1.5^g^ below the norm) on at most 1 test of executive functioning, social cognition, or language
(B) No evidence of decline from baseline (≥ 1.5 SD reduction on adjusted *Z* score) on any test of executive functioning, social cognition, or language

Adjusted *z*-score = *Z* score or equivalent standardised score (e.g. *T* score, index score), adjusted for age, sex, and education, where possible based on normative data

*MCI* mild cognitive impairment, *MCI-ft* frontotemporal-related mild cognitive impairment, *SubTCP* subthreshold cognitive performance, *SD* standard deviation

^a^In the presence of a relevant confound (e.g. depression, anxiety, significant psychosocial factor or event, disruption during testing), MCI-ft uncertain should be assigned

^b^Tests may be from the same or different cognitive domains (executive functioning, including letter fluency; social cognition; or language) but should assess at least two different cognitive processes (e.g. two executive functioning tests, with one primarily assessing inhibitory control and the other primarily assessing cognitive flexibility)

^c^On the ECAS, decline is determined based on published reliable change indices, where available, with the ALS-specific and Total scores counting as a single test of executive functioning and language

^d^Or ≥ 2 SD below equivalent standardised score (e.g. *T* score, index score)

^e^On the ECAS, ALS-specific and/or Total scores falling below version-specific published cut-offs count as subTCP on a single test of executive functioning, social cognition, and language

^f^ECAS is considered a single test of executive functioning, social cognition, and language, even if criteria are met for both decline and subTCP on the ECAS

^g^Or ≥ 1.5 SD below equivalent standardised score (e.g. *T* score, index score)

^h^Estimated using a standardised measure and incorporating the standard error

^i^Where only 1 test of executive functioning OR only 1 test of social cognition OR only 1 test of language OR only the ECAS has been administered, and there is no evidence of decline or subTCP

SubTCP or decline must be evident on two or more non-overlapping **tests** of executive functioning (including letter fluency), social cognition, or language (tests may capture the same or different domains).

### Characterising MCI-ft in Pre-fALS

The *Pre-fALS* dataset included 216 participants (94 *SOD1*, 74 *C9ORF72,* 20 other genes, and 28 non-carriers), with neuropsychological data at 807 visits (408 *SOD1*, 278 *C9ORF72*, 41 other genes, and 80 non-carriers). *SOD1* carriers had more visits than other genotypes, but there were no differences in baseline age, sex, or education (Table 1).

### Prevalence

MCI-ft was present at 4% of visits (4% *SOD1*, 5% *C9ORF72*, 7% other gene carriers, 1% non-carriers, Table 3). At a person level, 31/216 (14%) participants were classified as MCI-ft at one or more visits (13% *SOD1*, 20% *C9ORF72,* 15% other gene carriers, 4% non-carriers). Frequency and prevalence of MCI-ft classifications by genotype are summarised in Supplementary Table 4.

**Table 3:** Frequency and prevalence of MCI by different frameworks: *Pre-fALS* (All participants)

	MCI-ft (consensus review)^a^	MCI-ft (algorithmically derived)^b^	Revised Strong (algorithmically derived)^b^
Visit level			
*C9ORF72* (n = 278)	**15 (5%)**	18 (6%)	58 (21%)
*SOD1* (n = 408)	**16 (4%)**	18 (4%)	69 (17%)
*Other genes* (n = 41)	**3 (7%)**	2 (5%)	5 (12%)
Gene negative^c^ (n = 80)	**1 (1%)**	1 (1%)	8 (10%)
*TOTAL gene carriers* (n = 727)	***34 (5%)***	*38 (5%)*	*132 (18%)*
*TOTAL all participants* (n = 807)	***35 (4%)***	*39 (5%)*	*140 (17%)*
Person level			
*C9ORF72* (N = 74)	**15 (20%)**	16 (22%)	33 (45%)
*SOD1* (N = 94)	**12 (13%)**	12 (13%)	36 (38%)
*Other genes* (N = 20)	**3 (15%)**	2 (10%)	3 (15%)
Gene negative^c^ (N = 28)	**1 (4%)**	1 (4%)	4 (14%)
*TOTAL gene carriers* (N = 188)	***30 (16%)***	*30 (16%)*	*72 (38%)*
*TOTAL all participants* (N = 216)	***31 (14%)***	*31 (14%)*	*76 (35%)*

*n* number of visits, *N* number of participants

^a^Classifications following application of the MCI-ft Criteria and review by a research neuropsychologist, with consensus discussion as needed

^b^Classifications following application of the MCI-ft Criteria or Revised Strong Criteria algorithmically, without additional review or discussion

^c^Gene negative, i.e. unaffected individuals without a pathogenic variant associated with ALS or FTD

The bold values represent the the final prevalence values of MCI-ft in presymptomatic ALS as reported in the paper

The rows with italics are to highlight that these values represent subtotals/total of the information in the rows above (i.e. in visit-level, total gene carriers = C9ORF72 + SOD1+ Other genes and total all participants = C9ORF72 + SOD1+ Other genes + gene negative)

### The cognitive profile of MCI-ft in Pre-fALS

**Fig. 1:**
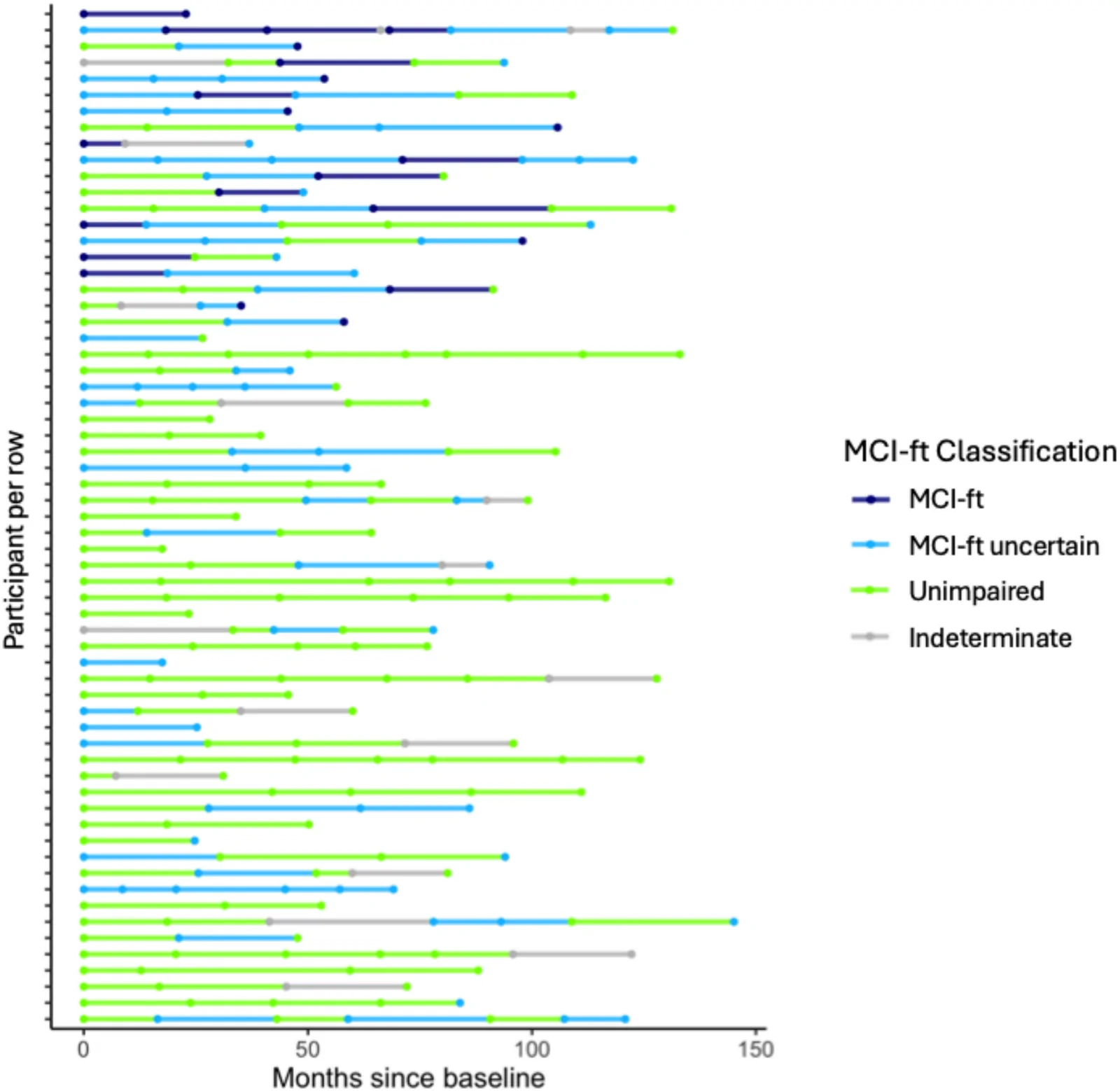
Changes in MCI-ft classification over time in a subset of 63 *Pre*-*fALS* participants. Each row represents a single participant, and each point represents a study visit. Each visit is classified based on the MCI-ft Criteria and reviewed by a research neuropsychologist, with consensus discussion as needed (colour coded in the legend). To ensure that data remains deidentified, a randomly selected subset of 63 *Pre-fALS* participants (known genetic status) have been included to illustrate fluctuations in MCI-ft status over time

Of the 35 visits that were classified as MCI-ft, 89% had impairment in executive functioning (including letter fluency which contributed to 40% of MCI-ft visits), 49% in language, and 6% in social cognition. Impairment on the ECAS ALS-specific or Total scores contributed to 37% of MCI-ft visits. Amongst the visits with MCI-ft, isolated subTCP alone was evident in 46% of visits, evidence of decline only in 23%, and both in 31%. Most visits were characterised by multi-domain impairment (24/35; 69%), with evidence of subTCP or decline on two or more tests of executive functioning, language, social cognition, or ECAS ALS-specific or Total score. The remaining visits with MCI-ft (11/35; 31%) showed subTCP or decline on tests capturing a single cognitive domain—most often executive functioning (31%). Since within-person fluctuations in MCI-ft classification over time were common (Fig. 1), we focus here on describing the cognitive profile of MCI-ft at a visit level.

Whilst subTCP or decline on ECAS alone is insufficient for MCI-ft, impairment on ECAS contributed to 37% of the visits with MCI-ft, with the remaining 63% of MCI-ft visits classified based on scores on the comprehensive neuropsychological evaluation only. There are two reasons that the ECAS may not capture impairment that is evident on comprehensive testing. First, some cognitive functions (e.g. semantic fluency or cognitive flexibility) are not captured by the ECAS. Amongst the 22 MCI-ft visits without impairment on the ECAS, 5 showed subTCP or decline on animal fluency, and 4 on the Stroop Test. The Wisconsin Card Sorting Test 64 item version [21] and the Multilingual Aphasia Examination Token Test [22], which were introduced in *Pre-fALS* in 2021, also captured impairment at 4 and 3 MCI-ft visits, respectively. Second, the ECAS uses summary scores encompassing tests of different cognitive domains, therefore lower performance on a specific task may be masked by performance within the expected range on other contributing tests. For example, impairment was evident on the letter fluency task and Boston Naming Test (BNT) [23] for 34% and 3% of MCI-ft designated visits, respectively, despite ECAS ALS-specific and total scores being unimpaired and unchanged from baseline.

### Comparison of algorithmically derived vs. reviewed MCI-ft status

Given that a review of all visit-level MCI-ft classifications by a research neuropsychologist or consensus group may not be feasible for all studies, we considered differences in algorithmically derived and consensus-designated MCI-ft status. An additional 23 visits were classified as MCI-ft algorithmically; of which, 22 were classified as MCI-ft uncertain and 1 as unimpaired with consensus review. Discordance in classifications was due to scores close to the cut-off for subTCP or decline from baseline (8 visits) or the presence of confounds (7 visits), which the algorithmically derived status does not account for. The other eight visits were algorithmically classified as MCI-ft largely due to the BNT—which has a high ceiling effect, such that an increase of just one or two errors represents a decline of ≥ 1.5 SD in the standardised score. All eight visits were classified as MCI-ft uncertain during consensus review.

### Comparison of the MCI-ft Criteria and Revised Strong Criteria

As cognitive status designation should not change based on whether an individual is in the presymptomatic or clinically manifest stage of ALS, we compared the prevalence of MCI using the MCI-ft and Revised Strong Criteria. The Revised Strong Criteria identified 17% (140/807) of visits as cognitively impaired and 35% (76/216) of individuals as cognitively impaired at any time point—significantly higher rates than identified through the MCI-ft Criteria (Table 3). Although 27/35 (77%) visits identified using the MCI-ft Criteria were also captured by the Revised Strong Criteria, the latter classified an additional 113 visits as cognitively impaired. The MCI-ft Criteria classified 100 (88%) of these visits as MCI-ft uncertain and 10 (9%) as unimpaired. The remaining three visits had only ECAS available, with no evidence of impairment or decline, so were classified as indeterminate based on the MCI-ft Criteria.

## Discussion

The overarching goal of this study was to advance understanding of MCI during the presymptomatic stage of ALS. First we examined the frequency of MCI using published frameworks for prodromal FTD, as well as presymptomatic and clinically manifest ALS [2, 5, 9] and found unexpectedly high and varying rates of impairment. This led us to examine the basis for these discordant rates of MCI and to refine the framework proposed following the 1st International Pre-Symptomatic ALS Workshop [9]. The resulting MCI-ft Criteria require evidence of decline (≥ 1.5 SD from baseline assessment) and/or subTCP (≥ 2 SD below norm) on two or more non-overlapping tests of executive functioning (including letter fluency), social cognition, or language (tests may capture the same or different domains). The MCI-ft Criteria also retain the “uncertain” research designation previously proposed [9], recognising that the interpretation of results of neuropsychological assessment may be unclear for various reasons. Separating MCI-ft from MCI-ft uncertain also helps prioritise specificity of the MCI-ft designation. We recommend that the MCI-ft Criteria be used in conjunction with consensus review, where possible, particularly for MCI-ft designated cases. Consensus review permits interpretation of the impact of confounds, or impairment on tests where data are not normally distributed (e.g. BNT). It also helps to ensure that classification is not based on a single cognitive function captured by the ECAS and comprehensive neuropsychological assessment. As such, the MCI-ft Criteria differs from the FTD community’s approach where a CDR® plus NACC FTLD score of 0.5 is intended to capture both those who are minimally impaired and those in whom impairment is questionable [24, 29].

Several aspects of the MCI-ft Criteria are worth highlighting. First, they focus on decline or subTCP on tests of executive functioning, social cognition, and/or language, excluding tests of memory and visuospatial skills. This contrasts with proposed frameworks for MCI in other neurodegenerative diseases which cover impairment in any major cognitive domain. Although subTCP on test of memory have been reported in ALS [25, 26] and FTD, whether this occurs independent of executive or attentional impairment continues to be debated [1]. Individuals presenting with isolated memory or visuospatial subTCP may best be categorised using established criteria for AD-related MCI.

Second, although the MCI-ft Criteria emphasise objective measures of cognitive decline, they also recognise subTCP without evidence of decline, which is helpful when repeated assessments have not yet been performed or are not possible. Whilst different cut-offs for subTCP have previously been proposed for MCI in ALS/FTD [2, 27], our findings suggest that a 2 SD cut-off minimises both: (1) over-classification of MCI-ft in non-pathogenic variant carriers, and (2) the risk that cognitive weaknesses, considered “normal” for the individual, are designated as representing MCI-ft. In these cases, it is possible that the onset of disease began early in life, or that cognitive subTCP may be unrelated to underlying ALS/FTD disease pathology.

Finally, the MCI-ft Criteria require a stringent definition of decline, emphasising objective cognitive change captured by comparing current scores to prior, typically baseline, performance. Whilst premorbid IQ and subjective cognitive decline (SCD) are often incorporated into criteria for MCI, poor reliability of existing measures at low and high premorbid IQ’s limits their interpretability. Additionally, standardised SCD measures are influenced by psychosocial factors [28], making SCD difficult to interpret in the absence of objective subTCP or decline. This may be particularly problematic in individuals who know their genetic status, as increased self-monitoring may make them more aware of subtle cognitive changes due to other factors (e.g., fatigue, mood). Based on these difficulties, the proposed MCI-ft Criteria include premorbid IQ and SCD as secondary sources of decline permitting, at most, a classification of MCI-ft uncertain. Additionally, although MCI criteria often consider functional abilities, the role of functional change in distinguishing prodromal FTD [5] from possible bvFTD [3] remains unclear [29]. Furthermore, few validated measures are available to assess functional changes associated with frontotemporal dysfunction, particularly in the presence of coexisting or emerging motor symptoms. Given these limitations, functional abilities were not incorporated into the proposed criteria.

Based on these refined criteria, MCI-ft was observed in 31 (14%) participants at 35 (4%) visits in the *Pre-fALS* study, with fluctuations in MCI-ft status over time. Visit-level prevalence may help inform cross-sectional screening and eligibility in clinical trials, whereas person-level estimates help to characterise the persistence, recurrence and potential relationship to phenoconversion. MCI-ft was more prevalent in *C9ORF72* and *SOD1* carriers (20% and 13%, respectively), compared to non-pathogenic variant carriers (4%). To the best of our knowledge, cognitive functioning in presymptomatic *SOD1* carriers has not previously been described. Recent research has challenged the view that affected *SOD1* carriers are cognitively unimpaired, showing poorer cognitive performance on tests of executive functioning [30]. However, these findings [30] were based on a small sample size (22 *SOD1* carriers and 10 *SOD1* non-carriers) and those who could not complete neuropsychological testing due to motor symptoms were excluded. Although not typically associated with FTD, the presence of MCI-ft in unaffected *SOD1* carriers may indicate an increased risk of phenoconversion to ALSci. Further examination of both presymptomatic and affected *SOD1* carriers will help determine whether the cognitive profile differs from *C9OR72* carriers and sporadic ALS patients.

Of those visits with MCI-ft, the majority had multi-domain involvement, with impairment across language, executive, and social cognition domains. These findings are consistent with previous research including 51 participants with ALS where language impairment (43%) and impairments in executive functioning (31%) were common [31]. Although lower average performance on letter fluency in presymptomatic *C9ORF72* gene carriers [10], affected ALS [32], and FTD [33] has been reported compared to controls, we could not identify any studies describing the frequency of letter fluency subTCP based on normative cut-offs in presymptomatic individuals. In clinically manifest ALS, prevalence has varied greatly (8–50% [25, 34, 35]). Despite accounting for motor symptoms, studies have been limited by small samples and differing subTCP definitions (e.g. based on published cut-offs vs. study control data), likely contributing to this variability.

Fluctuations in MCI status over repeated assessment were observed in the *Pre-fALS* study. Whilst similar fluctuation in MCI is reported in AD [36], fewer studies report longitudinal changes in CDR® plus NACC FTLD status. One study with baseline mild dementia (CDR-FTLD = 1, *n* = 68) showed that 14.7% were reclassified as prodromal 1 year later [37] suggesting that some fluctuation in classifications may also occur. However, this study [37] did not show individuals reverting from prodromal FTD (CDR-FTLD = 0.5, *n* = 84) to unimpaired. We were unable to identify specific reasons for fluctuations in the current study, and they did not appear to be due to varying performance on a specific test. Fluctuating status is not surprising, especially in the context of cognitive function where other factors (e.g. mood, fatigue) can impact performance. Alternatively, frequent fluctuations may indicate early disease instability. Future research should examine whether these fluctuations are associated with an increased risk of future phenoconversion to ALSci or FTD.

Whilst MCI-ft status might fluctuate over time, its designation at a single point in time should be independent of status on another axis (e.g. whether they are in the clinically silent, prodromal or clinically manifest stage of ALS). The proportion of visits identified as having cognitive impairment was higher based on the Revised Strong Criteria than the proposed MCI-ft Criteria. Using the MCI-ft Criteria pre-symptomatically and the Revised Strong Criteria after phenoconversion to ALS may, therefore, result in a “change” from no MCI-ft to ALSci, or vice versa, without any change in cognitive function. This would be problematic, especially in longitudinal studies. As our understanding of the prodromal stages of disease—including the development of subthreshold neuropsychological performance—improves, utilisation of the MCI-ft Criteria across the phenotypic continuum of the motor neuron axis might be considered.

Despite the proposed MCI-ft Criteria examining both executive and language domains together, whilst Strong et al. considers them separately, we observed higher rates of cognitive impairment using the Revised Strong Criteria compared to the MCI-ft Criteria. This likely reflects the fact that the Revised Strong Criteria permit classification of ALSci based on the ECAS alone [1], whereas the MCI-ft Criteria consider the ECAS scores as a single test, requiring subTCP or decline on another test of executive functioning, social cognition, or language to designate MCI-ft. These findings suggest that caution is warranted when applying the Revised Strong Criteria, developed for clinically manifest ALS, or relying on the ECAS alone, as this may result in an overestimation of MCI in presymptomatic disease. This also raises the question of whether the Revised Strong Criteria may be overly sensitive in clinically manifest ALS. Currently, the reliability of using ECAS in presymptomatic populations as a stand-alone measure of MCI is unknown. It is, however, important to note that, 63% of *Pre-fALS* visits classified as MCI-ft were based on comprehensive neuropsychological testing with unimpaired performance on the ECAS, with most of these classified based on tests that encompass cognitive functions not captured on the ECAS (e.g. cognitive flexibility, semantic fluency), highlighting the benefits of including more comprehensive testing to supplement the ECAS.

The Revised Strong Criteria also identified a higher-than-expected prevalence of MCI-ft in participants without a genetic risk of ALS (14%). Whilst it is possible that some of these participants may have AD-related cognitive impairment, it is unlikely given the focus on FTD-related cognitive domains, and the average age of *Pref-ALS* participants*.* However, family members of those who are carriers or affected with ALS/FTD may have an increased risk of ALS compared to those without a family history of either disease [38]. As such, they may not reflect the general population. Furthermore, although some studies have found lower cognitive scores, on average, amongst gene-negative family members compared to individuals without a family history of ALS [39], there is currently no evidence to suggest that this difference reflects a higher frequency of clinically meaningful cognitive impairments.

In the Revised Strong Criteria, social cognition is considered under the domain of executive functioning, reflecting evidence that these constructs overlap [2]. More recent evidence suggests that subTCP and poor performance on tests of social cognition may occur independently of deficits in executive functioning in ALS [40] and FTD [41, 42], and that social cognition is a more consistent early marker of FTD than executive dysfunction [43]. Based on this, and in keeping with the approach used by the FTD community [5], the MCI-ft Criteria recognise social cognition as a separate domain. Whilst social cognition is considered a cognitive domain, it overlaps with behaviours associated with FTD, including socially inappropriate behaviour and loss of sympathy and empathy. Whether impairments in social cognition contribute towards MCI-ft or MBI-ft status may therefore depend on the measure used (e.g. standardised tests of social cognition vs study partner report of observable behavioural changes).

The use of research “diagnostic” categories enables: a standardised approach in clinical research settings to facilitate between-study and within-study comparisons of prevalence or rates of cognitive impairment; and clear communication of “diagnosis” of MCI-ft, prognosis, and disease trajectory in research settings when appropriate [44]. Whilst more longitudinal data are needed to understand the predictive utility of an MCI-ft (and MBI-ft) designation, it is important to note that not all individuals will progress to FTD. Despite this, the MCI-ft and MBI-ft criteria are important in helping us study how cognitive and behavioural features develop, whether MCI-ft or MBI-ft differentially predict phenoconversion to ALS versus FTD, and to facilitate the study of the underlying biology of ALS and FTD. Whilst neurofilament light chain (NfL) has shown promise as a susceptibility/risk biomarker for ALS [45] and FTD [46], MCI-ft (and MBI-ft) status represent clinical syndromes that are defined independently of NfL concentration. Future investigations might explore whether MCI-ft/MBI-ft in combination with rising NfL strengthens our ability to predict the future risk of ALS/FTD phenoconversion.

## Conclusion

This analysis of extensive longitudinal cognitive data has enabled us to characterise the frequency and longitudinal course of MCI-ft during the presymptomatic stage of ALS; and to explore genotype-specific effects. The findings highlight the benefit of comprehensive neuropsychological assessment, in addition to established measures such as the ECAS, and consensus discussions to reliably detect MCI-ft. Reliably designating unaffected pathogenic variant carriers as having MCI-ft (or not) permits clear communication of MCI-ft status to research study participants when appropriate [44], empowers the study of biological mechanisms underlying MCI; and facilitates an evaluation of the prognostic utility of MCI in predicting future phenoconversion to clinically manifest disease. Improved ability to identify pathogenic variant carriers in the early stages of ALS/FTD should also enable enrolment in future early intervention trials that aim to delay or even prevent phenoconversion to clinically manifest disease.

## Supplementary Information

Below is the link to the electronic supplementary material.

Supplementary file1 (DOCX 105 KB)

## Data Availability

The datasets generated and/or analysed during the current study are not publicly available due to the need for additional precautions regarding the protection of confidentiality and blinding (especially for study participants in the non-disclosure group), but are available from the corresponding author (MB) on reasonable request and with a data use agreement in place.
