# Supplementary material for: Mild cognitive impairment in presymptomatic amyotrophic lateral sclerosis
Source: J Neurol. 2026 Oct 25;273(11):685. doi: 10.1007/s00415-026-14206-0 (PMC13696560; doi:10.1007/s00415-026-14206-0)
Supplement: Supplementary file 1 [file 415_2026_14206_MOESM1_ESM.docx]

# Supplementary Methods

## Comparison of Existing Criteria

Published frameworks for MCI in prodromal FTD, presymptomatic ALS, and clinically manifest ALS, were reviewed and summarised (Supplementary Table 1). To better understand the differences between frameworks, we used each to identify MCI in a randomly selected subset of asymptomatic *C9ORF72* carriers (n=32). The frequencies of cognitive impairment based on each framework were examined and compared.

We identified four key characteristics that differ between frameworks and might influence estimates of the frequency of MCI. These include: (1) the cognitive domains that should be considered (multi-domain vs ALS-FTSD specific domains); (2) the use of premorbid IQ to estimate decline in the absence of longitudinal data or to capture decline that might have occurred prior to baseline assessment; (3) different definitions of meaningful decline in scores over repeated assessment; and (4) the role of subTCP in the absence of decline (Supplementary Table 1).

## MCI-ft Criteria

We evaluated each characteristic to refine the MCI framework proposed following the 1^st^ International Presymptomatic ALS Workshop (WS1) [1] with additional refinements informed by practical issues relating to the application of the framework (e.g., assignment of MCI at a single assessment). The frequency of MCI based on each iterative variation of the framework was examined in *Pre-fALS* gene negative (i.e. control) participants, expecting, given how normative thresholds are conventionally set, that ~5% of individuals without a genetic risk of ALS/FTD might meet criteria for MCI. These findings informed modification or inclusion/exclusion of each key characteristic. This iterative process resulted in the MCI-ft Criteria.

# Supplementary Results

## Divergent rates of cognitive impairment based on published criteria

Three of the published frameworks[1–3] (Supplementary Table 1), were used to characterise a random subset of 32 *C9ORF72* carriers (known status or disclosure) with neuropsychological data from 127 study visits. Application of the remaining frameworks was limited, primarily due to ambiguities in published descriptions that made them difficult to operationalise. The subset of participants did not significantly differ from the remaining 42 *C9ORF72* carriers in the full *Pre-fALS* cohort in terms of age, sex, education, or number of study visits (data not shown). At the visit-level, the prevalence of cognitive impairment in the subset of 32 *C9ORF72* carriers was 12% based on the Revised Strong criteria, and 35% based on WS1. The MBCI-FTD criteria, which combines both cognition and behaviour, identified 45% of visits with impairment when using the version specified for pathogenic variant carriers.

At the person-level, the prevalence of cognitive impairment at any point during follow-up was discordant across frameworks and higher than expect - 28% (Revised Strong criteria), 66% (WS1), and 69% (MBCI-FTD). The three frameworks were also applied to 28 gene-negative controls (neuropsychological data at 80 visits) with person-level prevalences of 4% (MBCI-FTD version for non-pathogenic variant carriers), 10% (Revised Strong Criteria) and 14% (WS1). The WS1 was higher than the expected ~5%, indicating that it was likely too permissive.

## Evaluation of key characteristics that differed between frameworks

*Cognitive domains contributing to determination of cognitive decline*

The WS1 framework considered decline on tests of several cognitive domains including those commonly (executive functioning and language) and less commonly affected (memory and visuospatial skills) affected in ALS. However, published frameworks for prodromal FTD and clinically manifest ALS predominantly focus on executive (including letter fluency and social cognition) and language functioning. Restricting criteria to these domains may improve the specificity of the criteria for prodromal cognitive impairment related to ALS-FTSD. Furthermore, excluding decline on tests of memory and visuospatial functioning reduced the visit-level frequency of MCI in gene negative controls (Supplementary Table 3, V1).

*Premorbid IQ as benchmark for cognitive decline*

The WS1 framework included premorbid IQ to allow cross-sectional identification of MCI without requiring longitudinal assessment to indicate cognitive decline. Although estimated premorbid IQ is commonly used to interpret current performance,[4] available measures show poor reliability for low and high premorbid IQ’s.[5] Nevertheless, premorbid IQ may still be conceptually useful for flagging high functioning individuals where performance is lower than expected, and cognitive decline may have occurred prior to their first assessment. Excluding premorbid IQ as a means of identifying decline in executive functioning and language (when combined with exclusion of memory and visuospatial impairment) reduced the visit-level frequency of MCI to 0% in gene negative controls (Supplementary Table 3, V2).

*Threshold for defining meaningful decline*

The WS1 framework defined meaningful cognitive decline as a ≥ 1.5 standard deviations (SD) decline from baseline on age- and education-adjusted standardised score. Although prodromal FTD frameworks often also require evidence of decline, definitions vary or are not specified. Although using a less stringent definition of decline (1 SD) would improve the sensitivity of the criteria (Supplementary Table 3, V3), it would likely also increase the false-positive rate. While fluctuations in cognition have been associated with an increased risk of future dementia,[6] variability over repeated assessments may also occur due to stress, fatigue, changes in mood, and differing test-retest reliability of the measures.[7] To minimise the chance of false positive cases, a threshold of 1.5 SD was retained.

*SubTCP in the absence of objective meaningful decline*

The Revised Strong Criteria for cognitive impairment rely primarily on cross-sectional evidence of impairment (which we refer to here as subTCP) without requiring decline on tests of executive functioning, language or letter fluency. The rationale is that subTCP classification in the absence of decline permits designation of MCI based on an evaluation at a single point in time. We evaluated the proportion of visits classified as MCI based on evidence of subTCP *or* meaningful decline from baseline, using a 1.5 SD cut-off based on normative data. This increased the visit-level frequency of MCI in gene negative participants to 9%, almost double the expected level of ~5% (Supplementary Table 3, V4). Utilising a 2SD cut-off for subTCP resulted in a more acceptable prevalence of visits classified as MCI in gene negative participants (2%; Supplementary Table 3, V5)

*SubTCP and decline on letter fluency*

The Revised Strong Criteria and WS1 framework both permit classification of MCI based on subTCP and/or decline on a single test of letter fluency. This is based on evidence that people with ALS have lower average scores on letter fluency compared to controls [8]. Studies exploring the prevalence of subTCP on letter fluency tests in people with ALS have reported variable estimates, ranging from 8% to 50%. In addition, we are unaware of any studies examining the prevalence of subTCP on letter fluency tests in presymptomatic disease. Based on this, the final MCI-ft criteria takes a conservative approach in which subTCP or decline on a single test of letter fluency may contribute to the classification of MCI-ft, but is not sufficient in isolation. This resulted in a visit-level frequency of MCI-ft of 1% in gene negative participants (Supplementary Table 3, V6).

_._

**Supplementary Table 1. Summary of published frameworks for classifying cognitive impairment.**

| **Criteria** | **Developed for** |  | **Dysfunction** | **AND/OR** | **Decline** |
| --- | --- | --- | --- | --- | --- |
| ALSci (Strong et al., 2017) [2] | Clinically manifest ALS |  | Scores at or below the 5^th^ percentile on:   - 1 test of letter fluency OR - 2 tests of executive functioning ^a^ OR - 2 tests of language ^a^ | OR | 1.5 SD from baseline on:   - 1 test of letter fluency OR - 2 tests of executive functioning ^a^ OR - 2 tests of language ^a^ |
|  |  |  |  |  |  |
| WS1 (Benatar et al., 2022) [1] | Presymptomatic ALS |  | Not required. | - | From baseline OR premorbid IQ on:   - 1 test of letter fluency ^b^ OR - 2 non-letter fluency tests (executive functioning, language, memory, visuospatial) ^a,b^ |
|  |  |  |  |  |  |
| FT-MCI (de Mendonça et al., 2004) [9] | Prodromal FTD |  | 1 SD or below mean age and education adjusted z-scores on:   - 1test of executive functioning (attention, initiation, conceptual thinking). | - | Not required. |
|  |  |  |  |  |  |
| CDR plus NACC FTLD  (Knopman et al., 2008) [10] | Prodromal FTD |  | Score of 0.5 in any domain (memory; orientation; judgement and problem solving; community affairs; home and hobbies; personal care; behaviour comportment and personality; language) based on study-partner report or clinical evaluation. | AND | Based on study partner-report and/or clinical evaluation at a single timepoint, scores must represent cognitive loss from prior level rather than impairment due to other factors. |
|  |  |  |  |  |  |
| MBCI-FTD (Barker et al., 2022) [3] ^c^ | Prodromal FTD |  | 1.5 SD or below age, sex and education adjusted z-scores on:   - Executive functioning tests ^d,e^ OR - Naming (language) tests ^e^ | OR | Executive functioning OR Naming (language) tests ^f^ |
|  |  |  |  |  |  |
| MCBMI (Benussi et al., 2022) [11] ^c^ | Prodromal FTD |  | 1.5 SD below age, sex and education adjusted z-scores on executive functioning and/or language. ^e^ | AND | Self- or study partner reported, or clinically observed ^f^ |

WS1 – MCI framework proposed following the 1^st^ International Presymptomatic ALS Workshop; FT-MCI – frontotemporal mild cognitive impairment; CDR^®^ plus NACC FTLD – clinical dementia score plus National Alzheimer’s Coordinating Center Frontotemporal Lobar Degeneration; MBCI – mild behavioural cognitive impairment; MCBMI – mild cognitive behavioural motor impairment; MCI – mild cognitive impairment

^a^ Tests must not assess the same cognitive processes.

^b^ Based on reliable change indices, standardised regression-based formulas or standard deviation indices, as available.

^c^ Criteria combine cognitive impairment with other impairment (behavioural and/or motor). Cognitive impairment is not required to classify someone as prodromal. Details provided are for the cognitive component of the framework only.

^d^ Includes letter fluency.

^e^ Number of tests not specified.

^f^ Threshold to determine meaningful decline not specified.

# Supplementary Table 2. Neuropsychological assessments used in *Pre-fALS*

| **Cognitive domain** | **Cognitive process** | **Neuropsychological test** |
| --- | --- | --- |
| Premorbid IQ | - | North American National Adult Reading Test (NAART)[12] |
| Multi-domain | Multiple | North American Edinburgh Cognitive and Behavioural ALS Screen (ECAS)[13, 14] |
| Executive | Concept formation | Wisconsin Card Sorting Test – 64 item (WCST-64) [15] |
|  | Inhibition | Stroop Test |
| Social cognition | Theory of mind/Emotion recognition | Reading the Mind in the Eyes [16] Versions A and B [17] |
| Language | Naming | Boston Naming Test (BNT) [18, 19] |
|  | Comprehension | Token Test from the Multilingual Aphasia Examination (MAE) [20] |
|  | Semantic processing | Animal fluency |
| Letter fluency^a^ | Executive and language | FAS letter fluency |
| Processing speed | - | Symbol Digit Modality Test (SDMT) |
| Visuospatial | Object perception | Object Decision subtest from the Visual object and Space Perception Battery (VOSP) [21] |
|  | Spatial perception | Judgement of Line Orientation [22, 23] |
| Memory | Visual and verbal | Visual Reproduction subtest from the Wechsler Memory Scale-IV[24] |
|  | Immediate, delayed and recognition | California Auditory Verbal Learning Test (CVLT-II) [25] |

^a^ Letter fluency draws on both executive and language processes and therefore has been included here separately.

**Supplementary Table 3. Summary of different frameworks and rates of MCI – *Pre-fALS:* Gene negative participants**

|  | | **Variations** | | | | | |  |
| --- | --- | --- | --- | --- | --- | --- | --- | --- |
|  |  | **Original (WS1)** | **Cognitive domains considered**  **(V1)** | **Premorbid IQ as a benchmark for decline**  **(V2)** | **Threshold for meaningful decline from baseline**  **(V3)** | **SubTCP (1.5SD)**  **without decline**  **(V4)** | **SubTCP (2SD)**  **without decline**  **(V5)** | **MCI-ft Criteria**  **(V6)** |
| **Definitions for cognitive impairment** | **Incl Mem+VS** | Yes | No | No | No | No | No | No |
|  | **PremIQ Decline (SD)** | Yes (1.5) | Yes (1.5) | MCI uncertain ^a^ (1.5) | MCI uncertain ^a^ (1.5) | MCI uncertain ^a^ (1.5) | MCI-ft uncertain ^a^ (1.5) | MCI-ft uncertain ^a^ (1.5) |
|  | **Baseline Decline (SD)** | Yes (1.5) | Yes (1.5) | Yes (1.5) | Yes (1.0) | Yes (1.5) | Yes (1.5) | Yes (1.5) |
|  | **SubTCP (SD)** | MCI uncertain ^a^ (1.5) | MCI uncertain ^a^ (1.5) | MCI uncertain ^a^ (1.5) | MCI uncertain ^a^ (1.5) | Yes (1.5) | Yes (2.0) | Yes (2.0) |
|  | **Evidence on** | 2 tests ^b,c^  or LF | 2 tests ^b,c^  or LF | 2 tests ^b,c^  or LF | 2 tests ^b,c^  or LF | 2 tests ^b,c^  or LF | 2 tests ^b,c^  or LF | 2 tests ^b,c,d^ |
| **Visit-level** | **MCI** | 14 (18%) | 5 (6%) | 0 (0%) | 1 (1%) | 7 (9%) | 2 (2%) | 1 (1%) |
|  | **MCI uncertain** | 37 (46%) | 25 (31%) | 17 (21%) | 17 (21%) | 19 (24%) | 20 (25%) | 21 (27%) |
|  | **Unimpaired** | 26 (32%) | 47 (59%) | 60 (75%) | 59 (74%) | 51 (64%) | 55 (69%) | 55 (69%) |
|  | **Indeterminate** | 3 (4%) | 3 (4%) | 3 (4%) | 3 (4%) | 3 (4%) | 3 (4%) | 3 (4%) |

LF – letter fluency; Mem – memory; PremIQ – premorbid IQ; SD – standard deviation; SubTCP – Subthreshold cognitive performance; VS – visuospatial

WS1 – MCI framework proposed following the 1^st^ International Presymptomatic ALS Workshop

^a^ At most MCI uncertain

^b^ Two non-overlapping tests of language or executive functioning.

^c^ ECAS ALS specific score and ECAS total score counts as a single test.

^d^ Letter fluency counts as one test of executive/language functioning

**Supplementary Table 4. Frequency and prevalence of MCI-ft Criteria classifications** ^a^ **– *Pre-fALS* (All Participants)**

|  | **MCI-ft** | **MCI-ft uncertain** | **Unimpaired** | **Indeterminate** ^b^ |
| --- | --- | --- | --- | --- |
| ***Visit-level*** |  |  |  |  |
| *C9ORF72* *(n=278)* | 15 (5%) | 114 (41%) | 133 (48%) | 16 (6%) |
| *SOD1* *(n=408)* | 16 (4%) | 104 (25%) | 208 (75%) | 80 (20%) |
| Other genes *(n=41)* | 3 (7%) | 11 (27%) | 25 (61%) | 2 (5%) |
| Gene negative ^c^ *(n=80)* | 1 (1%) | 18 (23%) | 58 (72%) | 3 (4%) |
| *TOTAL gene carriers (n=727)* | *34 (5%)* | *229 (31%)* | *366 (50%)* | *98 (13%)* |
| *TOTAL all participants (n=807)* | *35 (4%)* | *247 (31%)* | *424 (53%)* | *101 (13%)* |
|  |  |  |  |  |
| ***Person-level*** |  |  |  |  |
| *C9ORF72* *(n=74)* | 15 (20%) | 42 (57%) | 17 (23%) | 0 (0%) |
| *SOD1* *(n=94)* | 12 (13%) | 45 (48%) | 36 (38%) | 1 (1%) |
| Other genes *(n=20)* | 3 (15%) | 8 (40%) | 9 (45%) | 0 (0%) |
| Gene negative ^c^ *(n=28)* | 1 (4%) | 12 (43%) | 15 (54%) | 0 (0%) |
| *TOTAL gene carriers (n=188)* | *30 (16%)* | *95 (51%)* | *62 (33%)* | *1 (<1%)* |
| *TOTAL all participants (n=216)* | *31 (14%)* | *107 (50%)* | *77 (36%)* | *1 (<1%)* |

^a^ Classifications following application of the MCI-ft Criteria and review by research neuropsychologist or consensus review therefore some frequencies and prevalences may differ from algorithmically-derived classifications.

^b^ Incomplete or no cognitive data available.

^c^ Gene negative, i.e., i.e. unaffected individuals without a pathogenic variant for ALS or FTD

n = Number of visits

N = Number of participants

Percentages may not equal 100% due to rounding.

**Supplementary references**

1.	Benatar M, Wuu J, McHutchison C, et al (2022) Preventing amyotrophic lateral sclerosis: insights from pre-symptomatic neurodegenerative diseases. Brain 145:27–44

2.	Strong MJ, Abrahams S, Goldstein LH, et al (2017) Amyotrophic lateral sclerosis - frontotemporal spectrum disorder (ALS-FTSD): Revised diagnostic criteria. Amyotroph Lateral Scler Frontotemporal Degener 18:153–174

3.	Barker MS, Gottesman RT, Manoochehri M, et al (2022) Proposed research criteria for prodromal behavioural variant frontotemporal dementia. Brain 145:1079–1097

4.	O’carroll R (1995) The Assessment of premorbid ability: A critical review. Neurocase 1:83–89

5.	Bright P, Hale E, Gooch VJ, et al (2018) The National Adult Reading Test: restandardisation against the Wechsler Adult Intelligence Scale—Fourth edition. Neuropsychol Rehabil 28:1019–1027

6.	Gamaldo AA, Allaire JC (2016) Daily fluctuations in everyday cognition: Is it meaningful? J Aging Health 28:834–849

7.	Heilbronner RL, Sweet JJ, Attix DK, et al (2010) Official position of the American Academy of Clinical Neuropsychology on serial neuropsychological assessments: the utility and challenges of repeat test administrations in clinical and forensic contexts. Clin Neuropsychol 24:1267–1278

8.	Abrahams S, Goldstein LH, Simmons A, et al (2004) Word retrieval in amyotrophic lateral sclerosis: a functional magnetic resonance imaging study. Brain 127:1507–1517

9.	de Mendonça A, Ribeiro F, Guerreiro M, Garcia C (2004) Frontotemporal mild cognitive impairment. J Alzheimers Dis 6:1–9

10.	Knopman DS, Kramer JH, Boeve BF, et al (2008) Development of methodology for conducting clinical trials in frontotemporal lobar degeneration. Brain 131:2957–2968

11.	Benussi A, Alberici A, Samra K, et al (2022) Conceptual framework for the definition of preclinical and prodromal frontotemporal dementia. Alzheimers Dement 18:1408–1423

12.	Blair JR, Spreen O (1989) Predicting premorbid IQ: A revision of the national adult reading test. Clin Neuropsychol 3:129–136

13.	Abrahams S, Newton J, Niven E, et al (2014) Screening for cognition and behaviour changes in ALS. Amyotroph Lateral Scler Frontotemporal Degener 15:9–14

14.	McMillan CT, Wuu J, Rascovsky K, et al (2022) Defining cognitive impairment in amyotrophic lateral sclerosis: an evaluation of empirical approaches. Amyotroph Lateral Scler Frontotemporal Degener 23:517–526

15.	Greve K (2001) The WCST-64: A standardized short-form of the Wisconsin card sorting test. Clin Neuropsychol 15:228–234

16.	Baron-Cohen S, Wheelwright S, Hill J, et al (2001) The “Reading the Mind in the Eyes” Test revised version: A study with normal adults, and adults with Asperger syndrome or high-functioning autism. J Child Psychol Psychiatry 42:241–251

17.	Burke T, Pinto-Grau M, Costello E, et al (2020) The reading the mind in the eyes test short form (A & B): validation and outcomes in an amyotrophic lateral sclerosis cohort. Amyotroph Lateral Scler Frontotemporal Degener 21:380–388

18.	Kaplan EF, Goodglass H, Weintraub S (1983) The Boston Naming Test, Second Edition. Lea & Febiger, Philadelphia

19.	Fisher N, Tierney M, Snow W, Szalai J (1999) Odd/Even short forms of the Boston Naming Test: preliminary geriatric norms. Clin Neuropsychol 13:359–364

20.	Benton AL, Des K, Sivan AB (1994) Multilingual aphasia examination, Third Edition. AJA associates

21.	Warrington EK, James M (1991) The Visual Object and Space Perception Battery: VOSP. Pearson

22.	Benton AL, Varney NR, Hamsher KD (1978) Visuospatial judgment: A clinical test. Arch Neurol 35:364–367

23.	Woodard JL, Benedict RH, Roberts VJ, et al (1996) Short-form alternatives to the Judgment of Line Orientation Test. J Clin Exp Neuropsychol 18:898–904

24.	Wechsler D (2009) Wechsler Memory Scale - (WMS-IV) technical and interpretive manual

25.	Delis DC, Kramer JH, Kaplan E, Ober BA (2000) California Verbal Learning Test - Second Edition, Adult Version. The Psychological Corporation, San Antonio, TX
